# Akutbelegungen in der Notaufnahme – was hat die COVID-19-Pandemie verändert?

**DOI:** 10.1007/s00063-024-01182-4

**Published:** 2024-09-26

**Authors:** Philipp Zehnder, Viktoria Bogner-Flatz, Michael Zyskowski, Frederik Hartz, Dominik Pförringer, Dominik Hinzmann, Karl-Georg Kanz, Michael Dommasch

**Affiliations:** 1 Klinik und Poliklinik für Unfallchirurgie, Klinikum rechts der Isar, Technische Universität München, München, Deutschland; 2 Klinik für Allgemeine, Unfall- und Wiederherstellungschirurgie, Klinikum der Universität München, Ludwig-Maximilians-Universität München, München, Deutschland; 3 Rettungszweckverband München, München, Deutschland; 4 Klinik für Anästhesiologie und Intensivmedizin, Klinikum rechts der Isar, Technische Universität München, München, Deutschland; 5 Zentrale interdisziplinäre Notaufnahme, Klinikum rechts der Isar, Technische Universität München, Ismaninger Str. 22, 81675 München, Deutschland

**Keywords:** Rettungsdienst, IVENA eHealth, Primärversorgung, Akutmedizin, Schockraum, Ambulance service, IVENA eHealth, Primary care, Acute medicine, Shock room

## Abstract

**Hintergrund:**

IVENA eHealth (IVENA, interdisziplinärer Versorgungsnachweis, mainis IT-Service GmbH, Offenbach am Main, Deutschland) unterstützt die Koordination von Notfallzuweisungen, indem es Echtzeitdaten über Behandlungsmöglichkeiten in Krankenhäusern liefert. Überlastung oder technische Probleme können dazu führen, dass Krankenhäuser Teile der Notaufnahme oder die gesamte Notaufnahme vorübergehend abmelden müssen, was zu Akutbelegungen führen kann. Die COVID-19-Pandemie könnte die Situation weiter verschärft haben, was Gegenstand dieser Untersuchung ist.

**Methodik:**

Diese deskriptive Analyse untersuchte mithilfe des IT-Systems IVENA eHealth die Belegungs- und Akutbelegungszahlen im Rettungsdienstbereich München von 2016 bis 2022. Besonderes Augenmerk lag auf den stationären Patienten (SK II) und den Zuweisungen von Schockraumpatienten + in den Fachgebieten Innere Medizin, Neurologie, Unfallchirurgie und Urologie sowie auf der Entwicklung der Akutbelegungen, insbesondere nach der COVID-19-Pandemie.

**Ergebnisse:**

Während der COVID-19-Pandemie im Jahr 2020 sank die Zahl der Patienten in den untersuchten Fachbereichen um 23,7 % (2021: −15 % und 2022: −11 % zu 2019). Der Anteil an Akutbelegungen sank 2020 im vgl. zu 2019 (5,9 % Akutbelegungen vs. 6,8 %) und stieg 2021 (7,7 % vs. 6,8 %) und 2022 (24,9 % vs. 6,8 %) überproportional an.

**Schlussfolgerung:**

Gründe für die Zunahme an Akutbelegungen sind vielfältig und umfassen unter anderen die Zunahme von stationären Einweisungen, den Engpass bei der Verlegung der Patienten (Exit-Block) sowie den Personalmangel im Gesundheitswesen. Die COVID-19-Pandemie verstärkte einige dieser Probleme, was die Zunahme an Akutbelegungen erklären könnte. Es bedarf jetzt einer Kombination verschiedener Lösungsansätze, um eine adäquate Notfallversorgung zu gewährleisten.

**Zusatzmaterial online:**

Zusätzliche Informationen sind in der Online-Version dieses Artikels (10.1007/s00063-024-01182-4) enthalten.

## Abstract (en)

**Background:**

IVENA eHealth (IVENA, interdisziplinärer Versorgungsnachweis, mainis IT-Service GmbH, Offenbach am Main, Germany) supports the coordination of emergency admissions by providing real-time data on treatment options in hospitals. Overload or technical problems can lead to hospitals having to temporarily deregister parts or the entire emergency department, which can lead to acute admissions. The COVID-19 pandemic may have further exacerbated the situation, which was analyzed as part of this study.

**Methods:**

This descriptive analysis used the IVENA eHealth information technology (IT) system to examine the occupancy and acute occupancy figures in the Munich ambulance service area from 2016–2022. Particular attention was paid to inpatient (SC II) and shock room + admissions in the specialties of internal medicine, neurology, trauma surgery and urology, as well as the development of acute occupancies, especially after the COVID-19 pandemic.

**Results:**

During the COVID-19 pandemic in 2020, the number of patients in the surveyed specialist areas fell by 23.7% (2021: −15% and 2022: −11% compared to 2019). The proportion of acute admissions fell in 2020 compared to 2019 (5.9% acute admissions vs. 6.8%) and rose disproportionately in 2021 (7.7% vs. 6.8%) and 2022 (24.9% vs. 6.8%).

**Conclusion:**

There are many reasons for the increase in acute admissions, including the increase in inpatient admissions, the bottleneck in transferring patients (exit block) and the shortage of staff in the healthcare system. The COVID-19 pandemic has exacerbated some of these problems, which could explain the increase in acute admissions. A combination of different solutions is now needed to ensure adequate emergency care.

Gründe für eine Akutbelegung in deutschen Notaufnahmen sind multifaktoriell. Die häufigsten Gründe sind hierbei eine Überbelastung oder z. B. technische Probleme. In den letzten Jahren ist die Zahl an Akutbelegungen stetig gestiegen. Inwiefern die COVID-19-Pandemie dies verändert hat, welche Ursachen dies haben könnte und was dies für unsere Patienten bedeutet, wird im Folgenden analysiert.

## Einleitung

„Die rund um die Uhr stattfindende Versorgung von Patienten mit akuten Gesundheitsstörungen ist eine unverzichtbare medizinische Dienstleistung für die Bevölkerung“, heißt es 2013 in einem Artikel von Behringer et al. zur Weiterentwicklung der Notfallmedizin in Deutschland, Österreich und der Schweiz [1]. In Deutschland fußt diese Notfallversorgung auf einem 3‑Säulen-Modell [2]. Die erste Säule bildet der ärztliche Bereitschaftsdienst organisiert durch die kassenärztliche Vereinigung, die zweite der Rettungsdienst und die dritte die Notaufnahmen der Krankenhäuser. Im Jahr 2019 wurden laut Zentralinstitut für die kassenärztliche Versorgung 10.272.213 ambulante und stationäre Fälle in deutschen Notaufnahmen behandelt [3]. Man geht davon aus, dass knapp die Hälfte der Patienten mit dem Rettungs- und Notarztdienst eingewiesen wird [4, 5]. In vielen Rettungsbereichen in Deutschland werden zur Koordination dieser Notfallzuweisungen digitale Anwendungen genutzt. Im Rettungsdienstbereich München werden seit Februar 2013 die Notfallzuweisungen mittels des IT-Systems IVENA eHealth (IVENA, interdisziplinärer Versorgungsnachweis, mainis IT-Service GmbH, Offenbach am Main, Deutschland) disponiert [6]. IVENA eHealth liefert für die jeweilige Rettungsleitstelle Echtzeitdaten über die aktuellen Behandlungsmöglichkeiten [7]. Der Rettungsleitstelle wird dabei angezeigt, ob das entsprechende Krankenhaus über ausreichend Kapazitäten verfügt, um einen entsprechenden Notfallpatienten zu behandeln. Die Krankenhäuser können bei Ausfällen von einzelnen Funktionsbereichen (z. B. Fachbereich Innere Medizin, Herzkatheter, Stroke-Unit etc.) den jeweiligen Teilbereich abmelden, sie können auch bei einer generellen Überlastung die gesamte Notaufnahme oder in Sonderfällen das gesamte Krankenhaus für einen gewünschten Zeitraum (1–24 h) abmelden. Gründe hierfür können technische Probleme, eine nichterfüllte Personaluntergrenze, Mangel an Behandlungsplätzen in der Notaufnahme (Overcrowding) oder ein sog. Exit-Block sein [8]. Ein Exit-Block liegt immer dann vor, wenn Notaufnahmepatienten aufgrund von fehlenden stationären Betten oder auch fehlenden Betten im Pflegeheim nicht abverlegt werden können [9]. Eine Abmeldung des entsprechenden Krankenhauses bedeutet jedoch nicht, dass dieses nicht mehr an der Notfallversorgung teilnimmt. Die Folge können sog. Akutbelegungen (ehemals Zwangsbelegungen) sein, also Belegungen eines Krankenhauses, das eigentlich nicht die strukturelle oder personelle Kapazität hat, diesen Patienten zu behandeln. Derartige Belegungen können für den Patienten und dessen Krankheitsverlauf schwerwiegende Folgen haben [10]. Die Häufigkeiten für derartige Akutbelegungen wurden 2020 von Rittberg et al. für die Jahre 2013 bis 2019 im Rettungsbereich München erfasst. Hier zeigte sich ein überproportional hoher Anstieg an Akutbelegungen (um den Faktor 9) bei einem Anstieg von 14,5 % der Gesamtzahl der Einsätze in diesem Zeitraum [11].

Ein bedeutender Faktor, der die Lage in den letzten Jahren weiter verschärft haben könnte, ist die COVID-19-Pandemie. Wie sich die Zahlen der Akutbelegungen im Rettungsbereich München in dem Pandemiejahr 2020 verhalten haben und insbesondere wie sich diese am Ende der Pandemie im Jahr 2022 entwickelt haben, ist Thema dieser Analyse.

## Methodik

Es handelt sich um eine retrospektive Analyse. Mithilfe des IT-Systems IVENA eHealth wurden die Belegungen und insbesondere die Akutbelegungen in den Jahren 2016 bis 2022 im Rettungsdienstbereich München analysiert.

Der Versorgungsbereich des Rettungsdiensts München umfasste im Jahr 2022 auf einer Fläche von 970 km^2^ 1.837.500 Menschen [12]. Eingeschlossen wurden alle Patienten, die über IVENA eHealth erfasst und in ein Krankenhaus eingeliefert wurden. Zusätzlich wurden die Fachgebiete „allgemeine Innere Medizin“, „Neurologie“, „Unfallchirurgie“ und „Urologie“ als Subgruppen analysiert. Hierbei lag der Fokus auf den stationären Zuweisungen (Dringlichkeits- bzw. Sichtungskategorie [SK] II), die mit ca. 80 % der Zuweisungen den größten Teil der Belegungen ausmachen. Außerdem wurde die Entwicklung der Akutbelegungen bei kritisch kranken bzw. schwerstverletzten Patienten, die eine Schockraumallokation (Schockraum +) notwendig machten, untersucht. Der Anteil dieser Patienten ist deutlich kleiner, bindet die meisten Ressourcen und benötigt in der Regel sofortige medizinische Maßnahmen. Ein besonderes Augenmerk lag auf den Veränderungen während und insbesondere nach der COVID-19-Pandemie. Hierzu wurden die Jahre 2019 und 2022 miteinander verglichen.

### IVENA eHealth

Seit Februar 2013 werden in München die Krankenhauseinweisungen durch den Rettungsdienst mittels des IT-Systems IVENA eHealth zugewiesen. Die Leitstelle kann anhand der Meldungen über die Kapazitäten und Verfügbarkeiten der beteiligten Krankenhäuser eine entsprechende ressourcenspezifische Disponierung durchführen. Die Krankenhäuser ihrerseits erhalten Meldung über eine Zuweisung inklusive geschätzter Ankunftszeit, Alter, Geschlecht und Zuweisungsgrund. Außerdem wird der durch den Rettungsdienst angeforderte Fachbereich (Augenheilkunde, Frauenheilkunde, Innere Medizin etc.) angegeben, das entsprechende Rettungsmittel genannt und die SK bestimmt. Hierbei wird zwischen SK I (Notfallversorgung), SK II (stationäre Versorgung) und SK III (ambulante Versorgung) unterschieden. Weiterhin werden auch die Schockraumanmeldungen über IVENA eHealth disponiert. Hierbei wird noch zwischen Schockraum − und Schockraum + unterschieden, wobei Schockraum + für die schwerer verletzten oder kritisch erkrankten Patienten steht.

### Statistik

Die Häufigkeiten werden als Anzahl oder/und in Prozent angegeben. Die Daten im Beobachtungszeitraum wurden anonymisiert aus IVENA eHealth übermittelt mittels Excel (Microsoft Excel 2019; Microsoft Office, USA) ausgewertet. Aufgrund der sehr hohen Fallzahl von 942.096 Patienten wurde auf die Berechnung von 95 %-Konfidenzintervallen verzichtet.

## Ergebnisse

Im Beobachtungszeitraum vom 01.01.2016 bis zum 31.12.2022 wurden in München insgesamt 942.096 Patienten mit dem Rettungsdienst in eine Klinik zugewiesen. Davon wurden 776.038 Patienten der Sichtungskategorie II (stationäre Versorgung) zugeordnet. Die Patienten waren zu etwas mehr als 50 % weiblich, es zeigten sich jedoch keine größeren Schwankungen hinsichtlich der Geschlechterverteilung zwischen den Jahren. Das Durchschnittsalter lag 2016 noch bei 56,8 Jahren und stieg im Jahr 2022 auf 60,6 Jahre an. Die Gesamtzahl der Klinikzuweisungen ist in den Jahren von 2019 bis 2022 um 3,2 % angestiegen (Tab. 1). Im ersten COVID-Jahr 2020 fiel die Zahl der Patienten in den untersuchten Fachbereichen von 92.622 auf 70.709 (Rückgang um 23,7 %). Im Verlauf stieg die Zahl der Patienten bis 2022 an, blieb jedoch weiterhin ca. 11 % unterhalb der Patientenzahl von 2019.

**Tab. 1:** Akutbelegungen nach Fachbereich

	2016	2017	2018	2019	2020	2021	2022
Patienten gesamt	131.719	131.750	137.454	141.939	127.201	132.389	139.644
Patienten SKII gesamt	111.658	110.157	115.039	116.673	103.437	106.997	112.077
Weibliches Geschlecht SK II	57.607	55.502	59.881	60.508	53.640	55.761	58.614
Weibliches Geschlecht in % SK II	51,6	50,4	52,1	51,9	51,9	52,1	52,3
Alter in Jahren SK II	56,8	57,9	58,5	59,5	60,9	60,6	60,6
Rettungsmittel KTW SK II	17.102	16.015	17.468	17.937	13.067	11.717	12.445
Rettungsmittel RTW SK II	84.035	84.096	86.769	88.925	81.691	86.573	90.911
Rettungsmittel NAW SK II	9273	8841	9889	9210	8075	8181	8095
Rettungsmittel RTH SK II	171	188	187	191	130	124	127
*Patientenanzahl SK II Innere, Unfallchirurgie, Neurologie, Urologie*	89.731	88.316	92.051	92.622	70.709	78.820	82.313
Davon Akutbelegungen	1040	2614	3257	6302	3605	6031	20.534
Davon Akutbelegung in %	1,2	3,0	3,5	6,8	5,1	7,7	24,9
*Allgemeine Innere Medizin SKII*	47.818	45.726	47.594	47.407	35.820	37.561	39.752
Davon Akutbelegungen	848	2245	2824	5114	2559	3622	14.358
Davon Akutbelegung in %	*1,77*	*4,91*	*5,93*	*10,79*	*7,14*	*9,64*	*36,12*
*Unfallchirurgie SKII*	33.113	33.671	34.402	34.860	24.955	30.365	32.104
Davon Akutbelegungen	60	29	72	101	56	104	664
Davon Akutbelegung in %	*0,18*	*0,09*	*0,21*	*0,29*	*0,22*	*0,34*	*2,07*
*Neurologie SK II*	5349	5418	6083	6251	5756	6302	6119
Davon Akutbelegungen	70	112	203	477	317	753	2053
Davon Akutbelegung in %	*1,31*	*2,07*	*3,34*	*7,63*	*5,51*	*11,95*	*33,55*
*Urologie SK II*	3451	3501	3972	4104	4178	4592	4338
Davon Akutbelegungen	62	228	158	610	673	1552	3459
Davon Akutbelegung in %	*1,8*	*6,5*	*4,0*	*14,9*	*16,1*	*33,8*	*79,7*
*Schockraum* *+*	2826	3093	3389	3962	3742	4497	4980
Davon Akutbelegungen	169	292	404	553	465	737	1279
Davon Akutbelegung in %	*5,98*	*9,44*	*11,92*	*13,96*	*12,43*	*16,39*	*25,68*

**Abb. 1:**
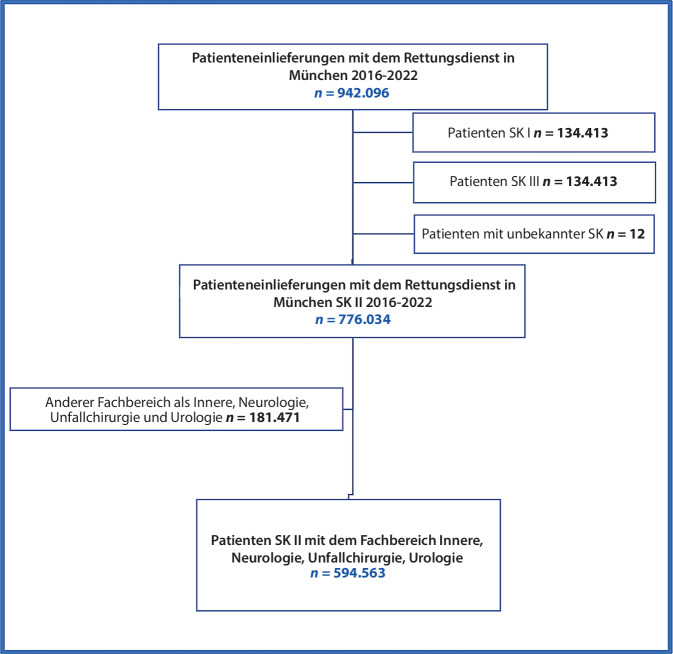
Flowchart

In den Fachbereichen „allgemeine Innere Medizin“, „Neurologie“, „Unfallchirurgie“ und „Urologie“ der Sichtungskategorie II wurden insgesamt 594.562 Patienten untersucht (Abb. 1). Dies entspricht einem Anteil von 76,6 % aller Patienten der Sichtungskategorie II.

**Abb. 2:**
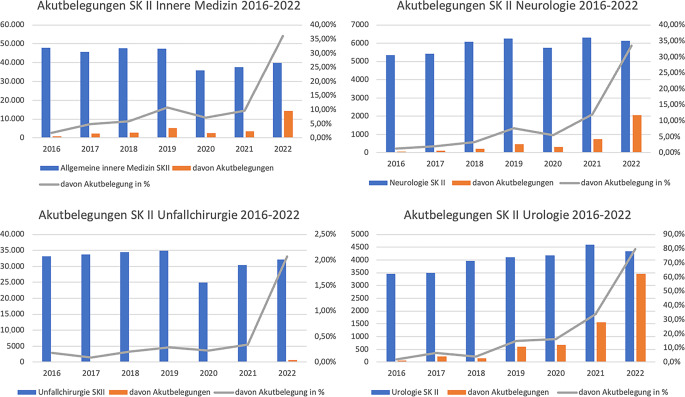
Anteil Akutbelegungen SK II nach Fachbereich 2016 bis 2022

In allen untersuchten Fachbereichen stieg der Anteil an Akutbelegungen von 2016 bis 2019 signifikant an (Tab. 1). 2020 war in 3 der 4 untersuchten Fachbereiche (Ausnahme Urologie) ein Rückgang der Akutbelegungen zu verzeichnen. 2022 zeigte sich ein deutlicher Anstieg in allen 4 Fachbereichen. Über den ausgewerteten Zeitraum von 2019 bis 2022 bedeutet dies eine Steigerung der Akutbelegungen im Fachbereich Innere Medizin um den Faktor 3,3; Neurologie um den Faktor 4,4; Unfallchirurgie um den Faktor 7,1 und Urologie um den Faktor 5,3. Insbesondere zeigt sich ein sprunghafter Anstieg in allen 4 untersuchten Fachbereichen von 2021 bis 2022 (Abb. 2).

**Abb. 3:**
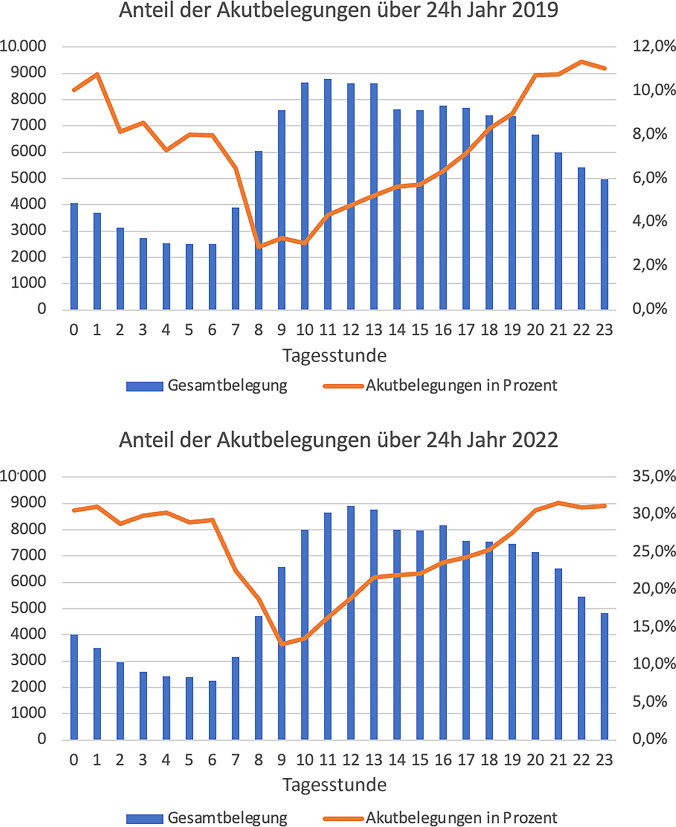
Anteil aller Akutbelegungen der Behandlungskategorie SK II über 24 h; 2019 und 2022

Gemittelt über den Tagesverlauf ist der prozentuale Anteil an Akutbelegungen in den Morgenstunden am geringsten. 2019 war der niedrigste Anteil an Akutbelegungen mit 2,9 % um 8.00 Uhr, 2022 zeigt sich der niedrigste Anteil an Akutbelegungen um 9.00 Uhr, allerdings mit einem Anteil von 12,7 % und damit um den Faktor 4,4 gesteigert. Über den Tagesverlauf kommt es zu einem Anstieg und 2019 wird der maximale Anteil an Akutbelegungen um 22.00 Uhr erreicht (11,3 %). 2022 steigt der Anteil der Akutbelegungen ebenfalls und erreicht um 21.00 Uhr mit 31,6 % den höchsten Wert (Abb. 3).

**Abb. 4:**
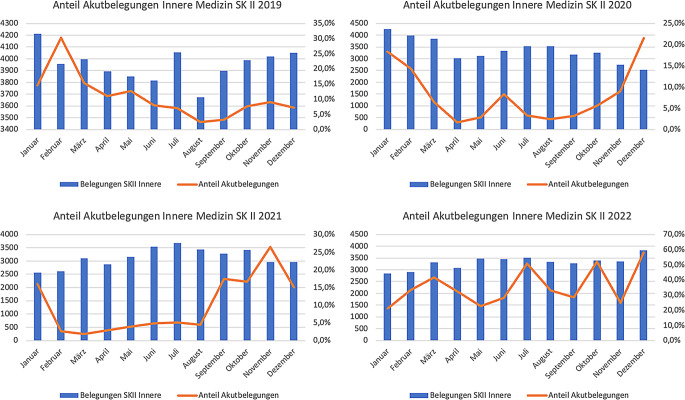
Anteil der Akutbelegungen Innere Medizin SKII über das Jahr; 2019 bis 2022

Betrachtet über das Jahr zeigt sich im Fachbereich „Innere Medizin“ ein saisonaler Unterschied in der Häufung der Akutbelegungen. Insbesondere in den Jahren 2019 bis 2021 ist ein Anstieg in den Wintermonaten zu verzeichnen, das Maximum wird in diesen Jahren zwischen den Monaten November und Februar erreicht. Im Jahr 2022 verhält sich die Verteilung anders und es zeigen sich mehrere Gipfel in der Monatsverteilung, z. B. im März, Juli, Oktober und November (Abb. 4).

Der Anteil an Akutbelegungen bei Patienten, die eine Schockraumbelegung (Schockraum +) benötigen, ist im Zeitraum 2016 bis 2019 von 6 % auf 14 % angestiegen. Es zeigte sich mit einem leichten „COVID-19-Knick“ (Rückgang um 11 %) ein kontinuierlicher Anstieg auf knapp 26 % Akutbelegungen im Jahr 2022 (siehe Abbildung im Zusatzmaterial online).

## Diskussion

Es handelt sich bei dieser deskriptiven Datenanalyse um eine der größten Auswertungen, die die Veränderung der Patientenzahlen in einem Rettungsdienstbereich nach und während der COVID-19-Pandemie untersuchte.

Von 2019 bis 2022 hat sich die Patientenanzahl wieder beinahe an das Vor-COVID-19-Niveau angenähert. So wurden 2022 knapp 140.000 Notfälle (2019: 141.000 ) durch den Rettungsdienst in Krankenhäuser eingewiesen. Im ersten COVID-Jahr 2020 war der Anteil der Notfallzuweisungen insgesamt um 10,4 % geringer als 2019. Betrachtet man die untersuchten Subgruppen (Innere Medizin, Neurologie, Unfallchirurgie, Urologie) in SK II, zeigte sich sogar ein Rückgang der Rettungsdienstzuweisungen um 23,7 %. Dies zeigten vergleichbar auch Müller et al. in ihrer Studie zum Rückgang der Rettungseinsätze im COVID-Jahr 2020 [13]. Mit dem Appell des Bundesgesundheitsministers am 13. März 2020 bündelten die Kliniken ihre Kapazitäten und nichtlebensbedrohliche Eingriffe wurden in der COVID-19-Pandemie verschoben [14]. So entstanden mehr freie Kapazitäten bei der Grundversorgung. Dies spiegelt sich auch in unseren Zahlen wider. So kam es bei rückläufigen Patientenzahlen zu einem deutlichen Rückgang der Akutbelegungen, verglichen mit den Vorjahren, in denen im Rettungsdienstbereich München sich die Akutbelegungen von 2013 bis 2019 fast verzehnfacht haben (Tab. 1). Ein weiterer Hinweis auf die Erhöhung der Kapazität und den damit verbundenen Rückgang der Akutbelegungen können die Daten zu den Schockraumpatienten liefern. Der Anteil der Akutbelegungen fiel um knapp 11 % bei einem beinahe konstanten Anteil an Schwerverletzten. In einer Analyse des deutschen Traumaregister war ebenfalls der Anteil an Schwerstverletzten während der COVID-19-Pandemie konstant [15].

Die Gründe für eine Zunahme an Akutbelegungen sind multifaktoriell. Zum einen wird die steigende Zahl an Selbsteinweisungen und damit an häufig ambulanten Patienten in der Notaufnahmen als Grund genannt [16]. Deutschlandweit verzeichnet das Zentralinstitut für die kassenärztliche Vereinigung bis 2016 auch einen deutlichen Anstieg der ambulanten Fälle. Diese nahmen allerdings seitdem bis zum 3. Quartal in 2020 wieder ab. Zum anderen nimmt die Zahl der stationären Einweisungen zu [3]. Die stationären Fälle sind hierbei häufig mit einem erhöhten Aufwand für die Notaufnahme verbunden. Als ein weiterer Grund für den Anstieg von Akutbelegungen wird häufig der Personalmangel, insbesondere im Bereich der Pflege, angeführt. In Deutschland fehlten bereits vor der Pandemie tausende Stellen, wie im Jahr 2018 im *Deutschen Ärzteblatt* ausgeführt wurde [17].

Im Jahr 2020 haben Rittberg et al. den Exit-Block, also die fehlende Möglichkeit der zeitnahen stationären Aufnahme von Notaufnahmepatienten, als eine Erklärungstheorie für die Zunahme an Akutbelegungen geliefert. Nur ein stetiger Patientenabfluss aus der Notaufnahme auf periphere Bettenstationen oder z. B. zurück ins Pflegeheim kann einen Exit-Block verhindern. Der ansonsten entstehende Rückstau von Patienten in die Notaufnahme führt letztlich zur Abmeldung bei der Leitstelle, was zu einer Zunahme an Akutbelegungen führt [11].

Ein Teil dieser Phänomene scheint sich durch die Pandemie noch verstärkt zu haben. Wir verzeichnen im Jahr 2022 beinahe 25 % Akutbelegungen innerhalb der untersuchten Sichtungskategorie und der entsprechenden Fachbereiche (2019 zum Vergleich 6,8 %). Dies hat zur Folge, dass jeder 4. Patient, der durch den Rettungsdienst in eine Notaufnahme eingeliefert wird, nicht ausreichend versorgt werden kann oder zumindest auf Strukturen trifft, die ggf. nicht ausreichen, um das akute Problem adäquat zu behandeln. Dies wiederum führt zu einer signifikant schlechteren Patientenversorgung und Abnahme der Patientensicherheit [10]. Man könnte vermuten, dass insbesondere die internistischen Fachbereiche unter der Pandemie gelitten haben, z. B. durch die große Anzahl der zu behandelnden Patienten und die belastenden Jahre. Allerdings verzeichneten wir auch zwischen den Jahren 2019 und 2022 innerhalb der Fachbereiche Unfallchirurgie (Anstieg um den Faktor 7), Neurologie (Anstieg um den Faktor 4,5) und Urologie (Anstieg um den Faktor 5) deutliche Anstiege an Akutbelegungen (siehe Abb. 2). Gleiches sehen wir bei Einweisungen in den Schockraum (Schockraum +). Jeder 4. Schockraumpatient wird im untersuchten Zeitraum in eine abgemeldete Klinik als Akutbelegung eingewiesen, also in eine Klinik mit mutmaßlich ausgeschöpften oder limitierten Ressourcen für die Behandlung von Schwerstverletzten bzw. -erkrankten.

Ein weiterer Erklärungsansatz für Akutbelegungen waren eine endemische Mehrbelastung durch eine Zunahmen an respiratorischen Erkrankungen und dadurch auch bedingte Personalausfälle, wie z. B. in der jährlich wiederkehrenden Influenzasaison [18]. Nach der COVID-19-Pandemie ist dieser saisonale Zusammenhang nicht mehr nachweisbar. Dies lässt vermuten, dass dieses nun ganzjährig auftretende Problem einen generellen Mangel an medizinischem Personal verursacht. Gründe hierfür können langfristige Erkrankungen (Burn-out) oder auch eine Neuorientierung weg von der Medizin sein [19, 20].

Eine Reduktion der „nichtdringlichen“ Patienten in Notaufnahmen wird gefordert. Dies reicht von der Bindung an einen Hausarzt bis zur Abweisung von Patienten durch die Notaufnahme hin in den ambulanten Sektor, wie jüngst vom Gemeinsamen Bundesausschuss (G-BA) beschlossen [21, 22]. Diese Umsetzung benötigt jedoch Zeit und außerdem zusätzliches, speziell geschultes Personal. Letztlich wurde dem G‑BA-Beschluss von Seiten des Bundesgesundheitsministeriums nicht zugestimmt und der Ausgang, ob ein solcher Beschluss in Umsetzung gebracht wird, ist noch offen.

Auch eine Steigerung der Gesamtkapazitäten der Notaufnahmen ist gefordert. Vorrangig soll der Anteil an Personal in den Notaufnahmen erhöht werden. Insbesondere eine Erhöhung der Bettenkapazität in den Notfallstationen und damit ggf. ein Abpuffern des Problems des Exit-Blocks sind in der Diskussion [23]. Dieses Problem wurde auch in der DGINA-Blitzumfrage im Jahr 2022 als dringlichstes Problem genannt. Die beteiligten Kliniken gaben an, dass aufgrund von Bettenmangel 28,5 % der Patienten in externe Kliniken verlegt werden mussten [8].

Mittelfristig könnte man zunächst die freien Kapazitäten, die bereits bestehen, besser nutzen. In Anlehnung an das Intensivregister der Deutschen Interdisziplinären Vereinigung für Intensiv und Notfallmedizin (DIVI) könnte man ein Register der freien Betten auf Normalstation entwickeln [24]. Politisch wurde dies bereits häufig gefordert, allerdings scheiterte dies insbesondere an der fehlenden Schnittstelle zwischen Krankenhaus und einem entsprechenden digitalen Informationssystem [25]. Um den Exit-Block in den Notaufnahmen zu vermeiden, sind „Bettenallokationssysteme“ sowohl klinikübergreifend als auch klinikintern ein probates Instrument. Patienten könnten zunächst in großen Notaufnahmen zügig eine aufwändige apparative Diagnostik erhalten und zur Weiterbehandlung entweder zeitnah im eigenen Haus oder in ein umliegendes Krankenhaus mit verfügbaren Kapazitäten abverlegt werden. Dies müsste allerdings finanziell für beide Krankenhäuser abbildbar sein bzw. zumindest nicht zu deren Nachteil sein.

Unsere Analyse zeigt einen klaren Trend mit einer deutlichen Zunahme an Akutbelegungen, die nach der COVID-19-Pandemie nicht mehr endemisch, sondern generell gehäuft auftreten. Das Ausmaß an Akutbelegungen ist unserer Ansicht nach als Surrogat für die strukturellen Probleme im Gesundheitswesen und im speziellen für die Akutversorgung von Patienten zu verstehen. Daher ist ein weiteres Aufschieben des Problems nicht zielführend und wahrscheinlich sogar patientengefährdend. Es benötigt eine Kombination aus mehreren Lösungsansätzen, die die Autoren leider auch nicht liefern können, um in Zukunft eine adäquate und dem Patientenaufkommen angepasste Notfallversorgung zu sichern.

## Limitationen

Es handelt sich hierbei um eine deskriptive Datenanalyse mit über 900.000 Patientendatensätzen mit entsprechenden Limitationen bedingt durch die beschriebene Form der Datenanalyse. Uns lagen lediglich Daten des Rettungsbereichs München vor. Buchungen anderer umliegender Rettungsdienstbereiche konnten nicht ausgewertet werden, sodass ein Teil der Rettungsdienstpatienten nicht erfasst wurde.

IVENA eHealth liefert keine expliziten Daten zur Schwere einer Erkrankung, weshalb diese aus dem Meldebild lediglich abgeschätzt werden konnte. Mit der Auswertung der SK-II-Patienten decken wir einen großen Teil der Patienten, die über den Rettungsdienst in eine Notaufnahme verbracht werden, ab. Wir haben jedoch bewusst, aufgrund der Heterogenität der Gruppen, auf die Auswertung von SK-I- und -III-Patienten verzichtet. Ferner fehlen uns auch die Gründe (Personalmangel, mangelnde Bettenkapazität etc.) für die Abmeldung der entsprechenden Notaufnahme, daher kann eine Aussage hierzu nur gemutmaßt werden. IVENA eHealth liefert zudem nur Daten der Patienten, die durch den Rettungsdienst eingewiesen wurden, die Zahlen der Gesamtpatienten der Notaufnahmen lagen uns nicht vor. Angaben und Auswertungen zum Gesamtaufkommen aller Notfälle in den Münchener Krankenhäusern waren daher mit dieser Analyse nicht möglich.

## Fazit für die Praxis

- Akutbelegungen haben in den letzten Jahren sukzessive zugenommen.
- Trotz rückläufigen Patientenzahlen hat dieser Trend während der COVID-19-Pandemie angehalten.
- Nach der COVID-19-Pandemie sind die Akutbelegungen überproportional gestiegen.
- Die hohen Akutbelegungen scheinen nach der COVID-19-Pandemie nicht mehr endemisch erklärbar zu sein, sondern werden vielmehr durch strukturelle Probleme verursacht.
- Ein weiteres Aufschieben des Problems ist nicht zielführend, sondern wahrscheinlich sogar patientengefährdend.

## Supplementary Information

Online Abbildung: Anteil der Akutbelegungen Schockraum +; 2016 bis 2022

## Data Availability

Die erhobenen Datensätze können auf begründete Anfrage in anonymisierter Form beim korrespondierenden Autor angefordert werden. Die Daten befinden sich auf einem Datenspeicher am TUM Universitätsklinikum, Klinikum rechts der Isar.
