# Supplementary figures and images for: Forced centralized allocation in the emergency department—what has the COVID-19 pandemic changed?
Source: Med Klin Intensivmed Notfmed. 2024 Sep 26;120(6):500–7. [Article in German] doi: 10.1007/s00063-024-01182-4 (PMC12411312; doi:10.1007/s00063-024-01182-4)

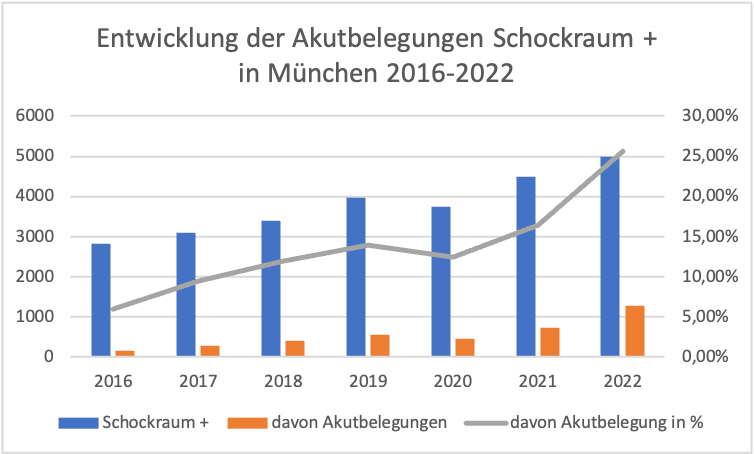


Online Abbildung: Anteil der Akutbelegungen Schockraum +; 2016-2022

Supplement: Supplementary file 1 — Online Abbildung: Anteil der Akutbelegungen Schockraum +; 2016 bis 2022 [file 63_2024_1182_MOESM1_ESM.docx]
